# PROGRESS (Patient-Reported Outcomes in Genital Reconstructive Surgeries): A Validated Patient-Reported Outcome Measure Questionnaire to Assess Post-Operative Functional Improvement Following Feminising Genital Reconstructive Surgery

**DOI:** 10.3390/jcm14082687

**Published:** 2025-04-14

**Authors:** Abi Kanthabalan, Feargus Hosking Jervis, Muhammad Hyder Junejo, Roland Morley, James Bellringer, Tina Rashid

**Affiliations:** 1 Chelsea and Westminster Hospitals, 369 Fulham Rd., London SW10 9NH, UK; tina.rashid1@nhs.net; 2 Urologistics Ltd., Ipswich IP4 2BF, UK; feargus@urologistics.com; 3 Department of Dermatology, Yale School of Medicine, New Haven, CT 06510, USA; 4 Imperial College Healthcare NHS Trust, London W2 1NY, UK; roland.morley@gmail.com; 5 Nuffield Health Parkside Hospital, London SW19 5NX, UK; james.bellringer@nhs.net

**Keywords:** feminising genital reconstructive surgery, PROMS questionnaire, validated questionnaire

## Abstract

**Background/Objectives**: Our aim was to validate a self-reported patient-reported outcome measure (PROM) questionnaire for use by patients undergoing feminising genital reconstructive surgery (fGRS). **Methods**: We used the Patient Reported Outcomes in Genital REconstructive SurgerieS (PROGRESS) questionnaire to examine key domains: urinary function, sexual function, cosmetic appearance, bowel function, and general health and wellbeing, which were identified as key components by our experienced surgeons. A reduction in score post-operatively represented an improvement in symptoms. Internal consistency was performed to determine the reliability of the questionnaire. **Results**: Between 2014 and 2024, 117 patients had completed pre- and post-operative questionnaires by week 52. The overall median score for all domains was 0.37 (interquartile range [IQR] 0.32–0.44) at baseline. At week 52 post-surgery, it was 0.24 (IQR 0.16–0.32), with a median difference (% change in overall score) of −0.12 (−35.8%) (*p* < 0.001). In all domains apart from bowel function, there was a reduction in scores that achieved statistical significance. There were high Cronbach’s alpha scores at baseline and at week 52 for four out of the five domains: general health and wellbeing a = 0.71 at week 0 and a = 0.79 at week 52; sexual function a = 0.83 at week 0 and a = 0.88 at week 52; cosmetic function a = 0.64 at week 0 and a = 0.84 at week 52; urinary function a = 0.74 at week 0 and a = 0.83 at week 52. Bowel function scored poorly, with a = 0.44 at week 0 and a = 0.49 at week 52. **Conclusions**: The questionnaire is suitable for use in clinical practice as a standardised way to assess functional outcomes following fGRS.

## 1. Introduction

The prevalence of transgender individuals in the UK is up to 1.2% in adults [1,2]. Individuals may experience gender dysphoria with high associated rates of functional impairment, self-harm, and suicide [1]. Surgery to align one’s genitals with a person’s chosen gender identity can often be the final stage of treatment and referrals to gender identity clinics are increasing yearly in the UK [1,2]. Our centre is part of a multi-disciplinary team offering feminising genital reconstructive surgery (fGRS) using various well-established techniques including vaginoplasty (penile skin inversion, free skin and tunica vaginalis grafts, pedicled scrotal flaps, and peritoneum and bowel segments) and vulvoplasty (also known as labioplasty or zero-depth vaginoplasty).

There is an absence of validated patient-reported outcome measures (PROMs) specific to genital surgery in the transgender population [3,4,5]. These systematic reviews report on the limited number and availability of postoperative questionnaires. Also, these all vary in terms of assessing different post-operative aspects: quality of life, female genital or sexual satisfaction, urinary function, aesthetic satisfaction, psychiatric, social, or psychosocial aspects, body image or gender dysphoria. These are all limited by either their development, their validation, or their content. PROMs questionnaires [3] can improve gender-affirming care through the regular monitoring of patient satisfaction (and facilitate studies of treatment effectiveness). PROMs can provide information and changes in patients’ symptoms, health-related quality of life, functional status, and satisfaction with care. PROMs developed by and for patients undergoing genital gender-affirming surgery are imperative to delivering high-quality patient-centred care [6]. The implementation of validated PROMs may guide shared decision-making by improving communication between surgeons and their patients; provide evidence and measurement of quality and standard of care received by patients; and inform service development and improvement [7]. PROMs questionnaires also have to be feasible and acceptable, i.e., easy to implement not burdensome to the patient or clinician and coherent to the patient [8,9,10]. The World Professional Association for Transgender Health has also emphasised the need for reliable, validated, and reproducible PROMs developed with patient involvement [11]. We report on our development and validation of a novel PROMS questionnaire: Patient-Reported Outcomes in Genital REconstructive SurgerieS (PROGRESS) for transgender patients.

## 2. Materials and Methods

### 2.1. Development of Questionnaire

The questionnaire was developed by expert clinicians (consultant surgeons performing fGRS and their specialist nurses) to examine key domains: urinary function, sexual function, cosmetic appearance, and general health and wellbeing. Pre-existing validated English-language instruments were reviewed for possible adaptation or application to a new instrument. Those thought particularly relevant were the International Prostate Symptoms Score (IPSS) and The Bristol Female Lower Urinary Tract Symptoms questionnaire for the urinary domain, the International Index of Erectile Function (IIEF) and the Female Sexual Function Index (FSFI) for sexual function, the Body Image Ideas Questionnaire (BIQ) for cosmetic appearance, and the Short Form 36 Health Survey Questionnaire (SF36) for general health and wellbeing. Content validity was determined by our expert team as each of the questions per domain was assessed to determine their validity.

Patient focus groups were held to determine if all important surgical aspects surrounding fGRS had been explored adequately and if the terminology used in the questionnaire was acceptable. As a result of the patient focus group, a domain was added to assess change in bowel function following fGRS. The Lower Anterior Resection Syndrome (LARS) Score was used for questions for this domain.

A score per question of 0 (least symptomatic) to 5 (most symptomatic) was calculated. A score of 5 represents a patient being most symptomatic in that domain. For example, if a patient scored 5 in answer to the question “What level of distress does your genitalia currently cause you?”, they are very distressed by their genitalia.

To determine which domain had the highest to least importance, six expert surgeons were given an arbitrary total sum of 15 points and then asked to rank the domains in order of most importance. The most important domain was allocated five points and the fifth was given one point. The number of points allocated to each domain was calculated and a percentage given (see Table 1); this was to allow weighting of each domain and as a result, the domains were ranked in order of importance as follows: general health and wellbeing (most important), sexual function, cosmetic appearance, urinary function, and lastly, bowel function (least important).

Participants were reviewed during clinic appointments at two different tertiary centres in the UK (Nuffield Health Parkside Hospital and Imperial College Healthcare NHS Trust). Baseline demographic data were recorded (Table 2) as part of best practice at the first visit. All participants were then sent a link to complete a secure, online questionnaire (which included a signed consent) prior to their surgery and then 10 and 52 weeks post operatively. The study was presented at local audit committee approval who deemed ethics was not required and research was completed in accordance with the Declaration of Helsinki as revised in 2013.

### 2.2. Statistical Analysis

The Wilcoxon signed-rank test was performed to compare improvement scores post-operatively. Internal consistency was assessed using Cronbach’s alpha statistic. REDCap version 12.2.5 was used for data collection and R 4.0.3 was used for analysis.

## 3. Results

### 3.1. Overall and Domain Scores

Between 2019 and 2021, 214 patients underwent fGRS across the two centres; 117 had completed pre- and post-operative questionnaires at week 52 at the time of our study. The time taken to complete the questionnaire was on average 9.63 min (median 10 min, standard deviation 7.1).

The overall median score for all domains was 0.37 (IQR 0.32–0.44) at baseline. At week 52 post-surgery, this score was 0.24 (IQR 0.16–0.32). There was a median difference (% change in overall score) of −0.12 (−35.8%) between week 0 and week 52 which achieved statistical significance (*p* < 0.001) and signified an overall improvement after surgery.

For sexual function, there was a median difference of −0.03 (−7.4%) between week 0 and week 52 which was statistically significant (*p* = 0.012) and signified an improvement in sexual function after surgery. For cosmetic appearance, there was a median difference of −0.47 (−77.9%) between week 0 and week 52 which was statistically significant (*p* < 0.001) and signified an improvement in cosmetic appearance after surgery. For urinary function, there was a median difference of 0.03 (+100%) between week 0 and week 52 which was statistically significant 0.03 (*p* < 0.001) and signified a worsening urinary function after surgery. Bowel function remained unchanged at 0 (0%) between week 0 and week 52. However, this was not statistically significant (*p* = 0.3).

For general health and wellbeing, there was a median difference of −0.07 (−28.8%) between week 0 and week 52 which was statistically significant (*p* = 0.001) and signified an improvement in general health and wellbeing after surgery.

We also analysed which symptom within each domain improved the most after surgery and which improved the least (or worsened) (Table 3, Table 4 and Table 5). Patients reported the greatest improvement in their ‘state of health and happiness’ in the general health and wellbeing domain and the one which worsened was their health state that day. In the sexual function domain, the symptom which was most improved after surgery was ‘an increase in sexual activity with vaginal penetration’ and the symptom which worsened was ‘experiencing pain in vagina/genitals unrelated to sexual activity’.

In the cosmetic function domain, the symptom which was most improved after surgery was ‘less avoidance of situations or activities such as swimming or changing in a public area’ and the one which worsened was ‘deliberately checking their genitals more’. In the urinary function domain, the symptom which was most improved after surgery was ‘decreased urinary urgency’; however, the symptom which worsened after surgery was ‘spraying of urine’. In the bowel function domain, the symptom which was improved the most after surgery was faecal urgency, but the symptom which improved the least was an increase in bowel movements.

### 3.2. Internal Consistency

Cronbach’s alpha is used to assess the validity and internal consistency of questionnaires with a score of α ≥ 0.9 reported as excellent, 0.9 > α ≥ 0.8 as good, 0.8 > α ≥ 0.7 as acceptable, 0.7 > α ≥ 0.6 as questionable, 0.6 > α ≥ 0.5 as poor, and α < 0.5 as unacceptable. Typically, a score of above 0.7 is deemed acceptable. Cronbach’s alpha was performed on each overall domain at both baseline and week 52 and then on questions within each domain.

Overall, there were high Cronbach’s alpha scores at baseline and at week 52: for general health and wellbeing α = 0.71 at week 0 and α = 0.79 at week 52, sexual function α = 0.83 at week 0 and α = 0.88 at week 52, cosmetic function α = 0.64 at week 0 and α = 0.84 at week 52, and urinary function α = 0.74 at week 0 and α = 0.83 at week 52. However, bowel function scored poorly, with α = 0.44 at week 0 and α = 0.49 at week 52, indicating that the bowel function questions may not be accurately assessing symptoms pre- and post-surgery (See Table 6).

Further analysis of Cronbach’s alpha was performed to determine if the score could be increased if a question per domain was removed to assess if the questionnaire could be made more relevant. For bowel function, the removal of one question (Question 43: Over the past 4 weeks, approximately how many times per day do you have a bowel movement?) would improve the alpha score from 0.485 to 0.606 at week 52.

## 4. Discussion

### 4.1. Comparison to Other Studies

Several PROMs questionnaires have been developed for both pre- and post-operative or medical therapy use. The International Prostate Symptom Score (IPSS) and The International Index of Erectile Function (IIEF) are examples of this. IPSS was developed for use in patients with benign prostatic hyperplasia [12]. Patients with clinically defined benign prostatic hyperplasia (BPH) were tested against control subjects who were unlikely to have BPH and in patients who underwent transurethral or open prostatectomy pre-operatively and at 4 weeks post-surgery. The questionnaire was found to have high accuracy in detecting improvement in symptoms following surgery. However, the study did not report on internal consistency in this cohort of patients.

The IIEF questionnaire is similar to our questionnaire as it has five key domains all of which have high internal consistency (α > 0.73 − 0.92). There were three parts to the study, with study A being most relevant to our comparison as it examined the improvement in erectile dysfunction symptoms after starting sildenafil. The IIEF was self-administered at the screening visit (week −4 or −2), at the end of the run-in phase (week 0), and at the end of 2, 4, 8, and 12 weeks of double-blind treatment. In patients who did respond to sildenafil, there was a score improvement in all domains which was detected by the IIEF with a high level of accuracy [13].

The low anterior resection syndrome score (LARS) questionnaire was examined on 961 patients [14]. However, re-test reliability was only examined in 29 patients who returned the questionnaire within 14 days and they did not use the questionnaire prior to their low anterior resection. The Kappa coefficient was also used for accuracy; however, this method of analysis only measures the accuracy of the questionnaire between time points and does not examine the inclusion or exclusion of questions to improve validity. This could explain the low internal consistency of this domain within the PROGRESS questionnaire.

The Bristol Female Lower Urinary Tract Symptoms questionnaire [15] was developed using the International Continence Society male questionnaire and validated in a small cohort of fifty patients. The questionnaire was conducted on pre- and post-urodynamics with a much shorter re-test of 2 weeks. The participants did not undergo any medical or surgical intervention. The questionnaire was found to have Cronbach’s α of 0.78. There was no clear psychometric benefit from subgrouping the items into categories of storage, voiding and incontinence; the Cronbach’s α for these groups were 0.48, 0.72 and 0.82, respectively, while the problem items had values of 0.69, 0.89, and 0.81, respectively. Reliability is essential if an instrument is to be of any use in clinical practice, and this appeared to be excellent for the questionnaire; both the internal consistency of the symptoms, as measured by Cronbach’s α, and the stability, as measured by test–retest analysis, were high.

The Body Image Ideals Questionnaire (BIQ) [16] examined female perspectives on ten physical attributes and asked them to score on what they deemed was their ideal and how well they matched this ideal (Discrepancy score), how important each ideal was (Importance score) and a weighted discrepancy score (mean of the 10 Discrepancy x Importance cross-products). These participants did not undergo any medical, surgical or psychological intervention. This questionnaire also had issues with scoring as the score of 0 was given as one option in the importance score. As a result, −1 was applied to any non-discrepant score. Cronbach’s α for Discrepancy, Importance, and the weighted discrepancy were 0.75, 0.82, and 0.77, respectively.

GENDER—Q [17] is a new phase I international study yet to be reported but consists of developing a PROMs questionnaire on issues that matter to transgender and gender diverse individuals namely experience of care, treatments and recovery, appearance, voice, psychological, body image, social, physical and sexual. It will be interesting to see the development and validity of the study and whether this is used at baseline and post-operatively on patients undergoing fGRS as several studies examining these domains are not validated in this cohort of patients.

Huber et al. [18] report on the development of a 33-item patient-reported outcome measure comprising appearance, urological, and gynaecologic domains in 61 patients at baseline and 2 weeks. The overall Cronbach’s α was 0.79, and for appearance, urological and gynaecological was 0.85, 0.87 and 0.42, respectively. This study, however, is a post-operative study and re-tests patients only 2 weeks later compared to our study that examines patient’s symptoms one year later with high internal consistency results in four out of five domains.

Our study included 117 patients which is greater than most major validating questionnaire studies described above. The PROGRESS questionnaire is one of the first questionnaires that has shown high validity and could be a useful tool for both patients and clinicians to assess pre- and post-operative functional outcomes in transgender patients undergoing fGRS.

### 4.2. Limitations

We accept that there are some limitations to our study. There is currently no gold standard questionnaire and therefore we cannot compare our questionnaire to assess for criterion validity. Each of the subdomain questions was taken from previously validated questionnaires. The questions from the general health and wellbeing domain were taken from the Short form 36 (SF36) health survey questionnaire [19], the questions from the sexual function domain were taken from The Female Sexual Function Index (FSFI) [20] and the IIEF Questionnaires [13]. Questions from the cosmetic function domain were taken from the BIQ [16]. Questions from the urinary function domain were taken from the IPSS [12] and Bristol Female Lower Urinary Tract Symptoms questionnaire [15] and questions from the bowel function domain were taken from the LARS Score [14].

Each of these questionnaires had a different number of possible responses. This posed a dilemma as it was felt the score must not over-represent sub-domains just because they have more questions, i.e., some questions have three responses and some have six responses. The score must not over-represent any particular response because of the number of responses provided to the participant (i.e., a score of 3/3 and 6/6 must be of equal value, and a score of 3/3 and 3/6 must not be of the same value). Secondly, there were options of “I don’t know” or “I do not wish to answer”. For this analysis, we excluded patients who did not answer all the questions at baseline and at Week 52. Where “I don’t know” or “I do not wish to answer” was provided as a response by the patient, it was not included in the calculation of the sub-domain. If a sub-domain has nine questions, but one question was unanswered, rather than take an average of the nine responses, an average was taken from the eight responses given.

Lastly, these questions differed in terms of scoring, i.e., a low score of 1 in urinary function actually reported a high level of symptoms “Over the past 4 weeks, how often have you had wetting of your pants during activities such as coughing, straining or jumping? 1—All the time, 5—Never”. This is in comparison to bowel function where a high score of 5 reported a high level of symptoms i.e., “Over the past 4 weeks, how often have you had any amount of accidental bowel leakage?” 5—All the time and 1 Never. It was felt that in order to assess symptoms appropriately these needed to be re-ordered to allow for a high score to show high patient distress and a low score to reflect low symptoms within the domains. The value of each response was then normalised to a value of between 0 and 1. The third most symptomatic response in a range of six would yield a normalised value of 0.5. The most symptomatic, 1, the least, 0. This gives equal weight to all questions regardless of the number of responses offered to the participant.

The value of each sub-domain was normalised to a value of between 0 and 1. If a sub-domain has nine questions, the average was taken from the nine responses. If a sub-domain has five questions, the average was taken from the five responses. As reported above, the weighting of sub-domains was taken based on the opinion of a group of UK Gender Reassignment Surgeons (UKGRS). Each of the five sub-domains was multiplied by their weighting factor. The average was then taken to generate the overall UKGRS score. There are issues with this as only six surgeons were asked to rank the domains. This could be considered a response bias as patients were also not asked to rank the domains they deemed from high to low importance.

In terms of clinical use, displaying the post-operative score to a patient in the clinic may have less of a visual impact and hence understanding when showing an improvement between 0 and 1 compared to a LIKERT score of 1–5. Whilst we have calculated a LIKERT score for patients (the score was multiplied by four, one was added, and then it was rounded to the nearest integer), we have not used it in the analysis or discussion in this paper as there is likely loss in accuracy due to the rounding of figures.

Another limitation was that our expert surgeons were asked to determine which domain had the highest to least importance. This could be a reflection of their preferred clinical outcomes; however, given their significant experience, factors reported by patients at follow up are likely to be the reason for ranking of each domain according to importance. It could be reasoned that the patient focus group should also have been consulted at the time to help rank the domains.

Lastly, bowel function was the subdomain that scored the least in terms of validity. Further analysis could involve the removal of the bowel function domain to see if the validity of the questionnaire can be improved. However, this domain was key to patients who wanted to report their bowel functions pre- and post-operatively.

## 5. Conclusions

The PROGRESS questionnaire is a self-administered questionnaire for the evaluation of the change in five key domains (general health and wellbeing, sexual function, cosmetic function, urinary function, and bowel function) experienced by patients who have undergone fGRS. This is the first such tool to be validated in transgender people who have undergone fGRS. We have shown an improvement in three out of the five key functional domains post-fGRS, (general health and wellbeing, sexual function, and cosmetic function) with some worsening of urinary function following surgery and no change in bowel function. The questionnaire has shown high internal consistency in four out of five domains; although, the assessment of the change in bowel function could be improved.

We would advocate for the use of the questionnaire as a standardised tool by clinicians who perform fGRS to objectively assess functional outcomes in their patients following fGRS.

## Figures and Tables

**Table 1:** Weighting of each domain.

	Surgeon 1	Surgeon 2	Surgeon 3	Surgeon 4	Surgeon 5	Surgeon 6
**1st Priority**	Sexual Function 5 points	General Health and Wellbeing 4.69 points	General Health and Wellbeing 3.26 points Cosmetic Appearance 3.26 points Sexual Function 3.26 points	General Health and Wellbeing 3.95 points Sexual Function 3.95 points	General Health and Wellbeing 5 points	Cosmetic Appearance 4.69 points
**2nd Priority**	Urinary Function 4 points	Sexual Function 3.75 points	Urinary Function 2.60 points Bowel Function 2.60 points	Cosmetic Appearance 3.16 points	Sexual Function 4 points	Urinary Function 3.75 points
**3rd Priority**	Cosmetic Appearance 3 points	Cosmetic Appearance 2.81 points		Urinary Function 2.37 points	Cosmetic Appearance 3 points	General Health and Wellbeing 2.81 points
**4th Priority**	General Health and Wellbeing 2 points	Urinary Function 1.88 points Bowel Function 1.88 points		Bowel Function 1.58 points	Urinary Function 2 points	Sexual Function 1.88 points Bowel Function 1.88 points
**5th Priority**	Bowel Function 1 point				Bowel Function 1 point	
	**15 points**	**15 points**	**15 points**	**15 points**	**15 points**	**15 points**

Each surgeon was given 15 points to allocate (5 + 4 + 3 + 2 + 1 = 15). The sum of the value of the choices they have made when the equal value is given to a sub-questionnaire (1st choice = 5, 2nd = 4 etc). Surgeon 2, above, has 5 + 4 + 3 + 2 + 2 = 16, surgeon 3 has 5 + 5 + 5 + 4 + 4 = 23. Divide the value given to each sub-questionnaire (1st choice = 5, 2nd choice = 4 etc) by (5 + 4 + 3 + 2 + 1)/sum for each surgeon. Surgeon 2′s 1st preference scores are 5 × (15/16) = 4.68. Surgeon 3′s 1st preference scores are 5 × (15/23) = 3.26. Please see Supplementary Materials.

**Table 2:** Participant summary.

Site	Imperial Healthcare NHS Trust	Putneymead Group
Median age	29.3 (IQR 25.7–43.4)	34.0 (IQR 27.1–46.5)
	*n*	*n*
Underweight	1	0
Healthy weight	15	3
Overweight	7	1
Ethnicity	*n*	*n*
White	43	2
Asian/Asian British	1	0
Unknown	1	0
NA	26	22

IQR, interquartile range; NA, not applicable; NHS, National Health Service.

**Table 3:** Difference in scores between week 0 and week 52.

	Score at Week 0	Score at Week 52	Median Difference	*p* Value
General health and wellbeing	0.24	0.14	−0.07	<0.001
Sexual function	0.56	0.47	−0.03	0.012
Cosmetic function	0.61	0.13	−0.47	<0.001
Urinary function	0.04	0.08	0.03	<0.001
Bowel function	0.15	0.17	0	0.3

**Table 4:** Responses with greatest improvement per domain.

Domain	Question	Median Difference
General Health and Wellbeing	Pick the statements that best describe your health state today with regards to your overall happiness…	−0.14
Sexual Function	Over the past 4 weeks, how often did you take part in sexual activity with vaginal penetration?	−0.21
Cosmetic Function	How often does your genitalia lead you to avoid situations or activities e.g., swimming, changing in a public changing area, being naked alone or in front of other people?	−0.76
Urinary Function	Over the past 4 weeks, how often have you had had to rush to get to the toilet to pass urine	−0.01
Bowel Function	Over the past 4 weeks, how often have you had an urgent need to empty your bowels that makes you rush to the toilet?	−0.02

**Table 5:** Responses with the most worsening of symptoms per domain.

Domain	Question	Median Difference
General Health and Wellbeing	Indicate on the scale to indicate how your health is TODAY	0.01
Sexual Function	Over the past 4 weeks, how often did you experience pain/unpleasant sensation in your vagina/genital area which was NOT related to sexual activity (including masturbation)?	0.09
Cosmetic Function	How often do you deliberately check your genitalia?	0.06
Urinary Function	Over the past 4 weeks, how often have you had spraying or a change in direction of your urine flow	0.28
Bowel Function	Over the past 4 weeks, how many times per day do you have a bowel movement?	0.08

**Table 6:** Cronbach’s alpha score at week 0 and week 52 per domain.

Domain	Cronbach’s Alpha Score at Week 0	Cronbach’s Alpha Score at Week 52
General health and wellbeing	0.71	0.79
Sexual function	0.83	0.88
Cosmetic function	0.64	0.84
Urinary function	0.74	0.83
Bowel function	0.44	0.49

## Data Availability

The original contributions presented in this study are included in the article. Further inquiries can be directed to the corresponding author.

## Supplementary Materials

The following supporting information can be downloaded at: https://www.mdpi.com/article/10.3390/jcm14082687/s1, File S1: PROGRESS Questionnaire.
