# Supplementary material for: PROGRESS (Patient-Reported Outcomes in Genital Reconstructive Surgeries): A Validated Patient-Reported Outcome Measure Questionnaire to Assess Post-Operative Functional Improvement Following Feminising Genital Reconstructive Surgery
Source: J Clin Med. 2025 Apr 14;14(8):2687. doi: 10.3390/jcm14082687 (PMC12027601; doi:10.3390/jcm14082687)
Supplement: Supplementary file 1 [file jcm-14-02687-s001.zip › jcm-3423401-supplementary.pdf]

PROGRESS Questionnaire

Urinary Function

Over the past 4 weeks, have you noticed a delay before you start to urinate?

All of the time
Most of the time
Sometimes
Occasionally
Never
I don't know/not sure
I don't wish to answer this question

Over the past 4 weeks, has the strength of your urinary stream been?

Reduced all of the time
Reduced most of the time
Reduced sometimes
Reduced occasionally
Normal
I don't know/not sure
I don't wish to answer this question

Over the past 4 weeks, have you ever had to strain to continue urinating?

All of the time
Most of the time
Sometimes
Occasionally
Never
I don't know/not sure
I don't wish to answer this question

Over the past 4 weeks, have you had to stop and start more than once while you urinate?

All of the time
Most of the time
Sometimes
Occasionally
Never
I don't know/not sure
I don't wish to answer this question

Over the past 4 weeks, how often have you felt your bladder has not emptied properly after you have urinated?

All of the time
Most of the time
Sometimes
Occasionally
Never
I don't know/not sure
I don't wish to answer this question

Over the past 4 weeks, how often have you had wetting of your pants during activities such as coughing, straining or jumping?

All of the time
Most of the time
Sometimes
Occasionally
Never
I don't know/not sure
I don't wish to answer this question

Over the past 4 weeks, how often have you had spraying or a change in direction of your urine flow?

All of the time
Most of the time
Sometimes
Occasionally
Never
I don't know/not sure
I don't wish to answer this question

Over the past 4 weeks, how often have you had had to rush to get to the toilet to pass urine?

All of the time
Most of the time
Sometimes
Occasionally
Never
I don't know/not sure
I don't wish to answer this question

Over the past 4 weeks, how often have you had a urinary tract infection which needed treatment with antibiotics?

None
Once
Twice
Three times
Four or more times
I don't know/not sure
I don't wish to answer this question

Sexual Function

Over the past 4 weeks, how often have you felt sexual desire?

Almost never/never
A few times
Sometimes
Most times
Almost always/always
I don't know/not sure
I don't wish to answer this question

Over the past 4 weeks, how would you rate your level of sexual desire?

Very low or none at all
Low
Moderate
High
Very high
I don't know/not sure
I don't wish to answer this question

Over the past 4 weeks, how often did you have a feeling of sexual arousal in your genital area when you took part in sexual activity (including masturbation)?

Not at all
Sometimes
Often
Very often
Every time
I don't know/not sure
I don't wish to answer this question
I did not take part in sexual activity

Over the past 4 weeks, how much sexual arousal did you feel in your genital area when you took part in sexual activity (including masturbation)?

None
Slightly warm
Moderately warm
Very warm
Extremely warm
I don't know/not sure
I don't wish to answer this question
I did not take part in sexual activity

Over the past 4 weeks, during sexual activity (including masturbation), how often did you achieve self-lubrication?

Almost never/never
A few times
Sometimes
Most times
Almost always/always
I don't know/not sure
I don't wish to answer this question
I did not take part in sexual activity

Over the past 4 weeks, how often did you have an orgasm when you took part in sexual activity (including masturbation)?

Not at all
Sometimes
Often
Very often
Every time
I don't know/not sure
I don't wish to answer this question
I did not take part in sexual activity

Over the past 4 weeks, how pleasurable were the orgasms that you had?

Not pleasurable
Slightly pleasurable
Moderately pleasurable
Very pleasurable
Extremely pleasurable
I don't know/not sure
I don't wish to answer this question
I did not have any orgasms

Over the past 4 weeks, how easy was it for you to reach orgasm?

Very difficult
Quite difficult
Neither easy nor difficult
Quite easy
Very easy
I don't know/not sure
I don't wish to answer this question
I did not have any orgasms

Over the past 4 weeks, how often did you experience pain/unpleasant sensation in your genital area during or up to 30 minutes after sexual activity (including masturbation)?

Not at all

Sometimes
Often
Very often
Every time
I don't know/not sure
I don't wish to answer this question
I did not take part in sexual activity

Over the past 4 weeks, how much pain did you experience in your genital area during or after sexual activity (including masturbation)?

Not painful
Slightly painful
Moderately painful
Very painful
Extremely painful
I don't know/not sure
I don't wish to answer this question
I did not take part in sexual activity

Over the past 4 weeks, how often did you experience pain/unpleasant sensation in your genital area which was NOT related to sexual activity (including masturbation)?

Not at all
Sometimes
Often
Very often
Every time
I don't know/not sure
I don't wish to answer this question

Over the past 4 weeks, how often did you feel emotionally close to your partner when you took part in sexual activity (including masturbation)?
Please leave this question blank if you do not have a partner

Not at all
Sometimes
Often
Very often
Every time
I don't know/not sure
I don't wish to answer this question
I did not take part in sexual activity

Over the past 4 weeks, how confident have you felt about yourself as a sexual partner?

Not at all

Slightly
Moderately
Very
Extremely
I don't know/not sure
I don't wish to answer this question

Over the past 4 weeks, how satisfied have you been with your overall sex life?

Very dissatisfied
Moderately dissatisfied
Equally satisfied and dissatisfied
Moderately satisfied
Satisfied
Very satisfied
I don't know/not sure
I don't wish to answer this question

Cosmetic Appearance

How often do you deliberately check your genitalia?

About 40 or more times a day
About 20 times a day
About 10 times a day
About 5 times a day
Never
I don't wish to answer this question

How do you feel about the appearance of your genitalia?

Very attractive
Attractive
Slightly unattractive
Moderately unattractive
Markedly unattractive
Extremely unattractive
I don't wish to answer this question

What level of distress does your genitalia currently cause you?

Not at all
Slightly
Moderately
Markedly
Extremely
I don't wish to answer this question

How often does your genitalia lead you to avoid situations or activities e.g. swimming, changing in a public changing area, being naked alone or in front of other people?

Always avoid
Avoid ¾ of the time
Avoid half the time
Avoid ¼ of the time
Never avoid
I don't wish to answer this question

How much does your genitalia currently preoccupy you? This is, you think about it a lot and it is hard to stop thinking about it?

Not at all
Slightly
Moderately
Markedly
Extremely
I don't wish to answer this question

If you have a partner, how much does your genitalia currently have an effect on your relationship with an existing partner?
If you do not have a partner, how much does it have an effect on dating or developing a relationship?

Not at all
Slightly
Moderately
Markedly
Extremely
Not Relevant
I don't wish to answer this question

How much does the appearance of your genitalia currently interfere with your ability to work or study, or your role as a homemaker?
Please rate this even if you are not working or studying: we are interested in your ability to work or study.

Not at all
Slightly
Moderately
Markedly
Very severely, I can't work
I don't wish to answer this question

How much do your feelings about the appearance of your genitalia currently interfere with your social life?

Not at all
Slightly
Moderately
Markedly
Totally
I don't wish to answer this question

How much do you feel the appearance of your genitalia makes you feel complete?

Not at all
Slightly
Moderately
Mostly
Totally
I don't wish to answer this question

How inconvenienced are you by measures to accommodate your genitalia, e.g. tucking, specific underwear, use of pads, use of tape?

Not at all
Slightly
Moderately
Markedly
Totally
I don't wish to answer this question

Bowel Function

Over the past 4 weeks, approximately how many times per day do you have a bowel movement?

Less than once a day
Once a day
More than once a day

Over the past 4 weeks, how often have you had any amount of accidental bowel leakage?

Never
Occasionally
Sometimes
Most of the time
All of the time
I don't know/not sure
I don't wish to answer this question

Over the past 4 weeks, how often have you had an urgent need to empty your bowels that makes you rush to the toilet?

Never
Occasionally
Sometimes
Most of the time
All of the time
I don't know/not sure
I don't wish to answer this question

Over the past 4 weeks, how often have you been constipated?

Never
Occasionally
Sometimes
Most of the time
All of the time
I don't know/not sure
I don't wish to answer this question

Over the past 4 weeks, how often have you had diarrhoea?

Never
Occasionally
Sometimes
Most of the time
All of the time
I don't know/not sure
I don't wish to answer this question

General Health and Wellbeing

For each question, please pick the statements that best describe your health state today

With regards to work:

I have no problems with performing my usual activities
I have some problems with performing my usual activities
I am unable to perform my usual activities
I don't wish to answer this question

With regards to exercise/leisure activities

I have no problems with performing my usual activities
I have some problems with performing my usual activities

I am unable to perform my usual activities
I don't wish to answer this question

With regards to your overall happiness

I am mostly happy
I am equally happy and unhappy
I am mostly unhappy
I don't wish to answer this question

With regards to anxiety/depression

I am not anxious or depressed
I am moderately anxious or depressed
I am extremely anxious or depressed
I don't wish to answer this question

We would like to know how good or bad your health is TODAY

This scale is numbered from 0 to 100
100 means the best health you can imagine
0 means the worst health you can imagine

Score ___
